# High rate and stable cycling of lithium metal anode

**DOI:** 10.1038/ncomms7362

**Published:** 2015-02-20

**Authors:** Jiangfeng Qian, Wesley A. Henderson, Wu Xu, Priyanka Bhattacharya, Mark Engelhard, Oleg Borodin, Ji-Guang Zhang

**Affiliations:** 1 Joint Center for Energy Storage Research; 2 Energy & Environment Directorate, Pacific Northwest National Laboratory, Richland, Washington 99352, USA; 3 Environmental and Molecular Sciences Laboratory, Pacific Northwest National Laboratory, Richland, Washington 99352, USA; 4 Electrochemistry Branch, Sensor & Electron Devices Directorate, U.S. Army Research Laboratory, Adelphi, Maryland 20783, USA

## Abstract

Lithium metal is an ideal battery anode. However, dendrite growth and limited Coulombic efficiency during cycling have prevented its practical application in rechargeable batteries. Herein, we report that the use of highly concentrated electrolytes composed of ether solvents and the lithium bis(fluorosulfonyl)imide salt enables the high-rate cycling of a lithium metal anode at high Coulombic efficiency (up to 99.1%) without dendrite growth. With 4 M lithium bis(fluorosulfonyl)imide in 1,2-dimethoxyethane as the electrolyte, a lithium|lithium cell can be cycled at 10 mA cm^−2^ for more than 6,000 cycles, and a copper|lithium cell can be cycled at 4 mA cm^−2^ for more than 1,000 cycles with an average Coulombic efficiency of 98.4%. These excellent performances can be attributed to the increased solvent coordination and increased availability of lithium ion concentration in the electrolyte. Further development of this electrolyte may enable practical applications for lithium metal anode in rechargeable batteries.

## Abstract (web summary)

Lithium metal is an ideal anode material for rechargeable batteries, but lithium dendritic growth and limited Columbic efficiency prevent its applications. Here, the authors report the use of highly concentrated electrolytes composed of ether solvents and the salt lithium bis(fluorosulfonyl)imide to enable high-rate cycling of lithium anode.

## Graphical abstract

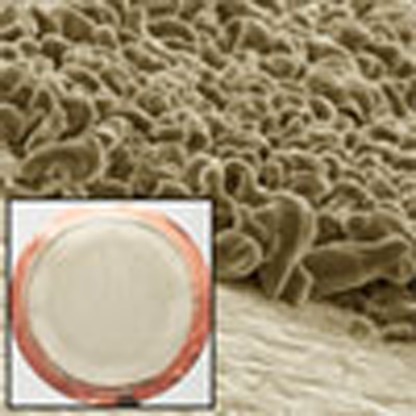

Lithium (Li) metal is an ideal anode material for rechargeable Li batteries due to its extremely high theoretical specific capacity (3,860 mAh g^−1^), low density (0.534 g cm^−3^) and the lowest negative electrochemical potential (−3.040 vs standard hydrogen electrode). Extensive attempts have been made to use Li as an anode in rechargeable Li batteries since the 1970s^1^, but several seemingly insurmountable barriers, including dendritic Li growth and limited Columbic efficiency (CE) during repeated Li deposition/stripping processes, have prevented their large-scale applications^2^,^3^,^4^. Since Sony and Asahi Kasei released the first commercial Li-ion batteries in 1991 using graphite to replace Li metal as the anode, almost all commercial Li-ion batteries have used various forms of carbon as their anode materials^5^. However, after more than 20 years of development, the energy density of graphite-based Li-ion batteries may eventually reach their limit in the near future^1^. Therefore, efforts to use Li metal as the anode for rechargeable Li batteries (such as Li-S and Li-air batteries) have revived in recent years, as Li metal has a theoretical capacity ten times as high as that of graphite^6^.

Electrolyte is one of the most critical elements that affects the cycling stability of Li metal anodes. Aurbach *et al.*^4^ indicated that Li is thermodynamically unstable with any kinds of organic solvents. The interactions between electrolyte components and Li metal results in significant side reactions that not only lead to a low CE but also consume Li metal and the electrolyte. This produces a solid-electrolyte interphase (SEI) film that may eventually grow into a thick layer, leading to high-impedance-failure of the battery instead of a short circuiting failure due to dendritic Li growth^7^. This phenomena becomes serious especially at high current densities^7^. Therefore, extensive studies have been conducted to understand how electrolyte formulations affect the cycling of Li metal electrodes^2^,^7^,^8^,^9^,^10^,^11^. Organic carbonate solvents and LiPF_6_ salt have been widely used in Li-ion batteries due to their wide electrochemical stability windows and good compatibility with conventional intercalation electrodes. However, it is well established that the plating/stripping efficiency of Li metal in electrolytes with carbonate solvents such as propylene carbonate (PC) is poor and typically results in dendritic Li metal deposits and a low CE of only <80%^9^,^12^. These carbonate solvents can be reduced during Li deposition process and form Li alkyl carbonate (ROCO_2_Li) species and, if trace water is present, may further react to form Li_2_CO_3_ (refs 3, 13, 14). The SEI layers dominated by these components are usually not strong enough to accommodate the rapid changes in the morphology of the plated Li; hence, Li dendrites readily penetrate the SEI layer and lead to battery short circuits. The use of additives (for example, vinylene carbonate (VC), fluoroethylene carbonate (FEC) and so on) and/or replacement of the LiPF_6_ electrolyte salt with other salts—such as LiAsF_6_, LiBF_4_, LiClO_4_, LiCF_3_SO_3_ or LiTFSI (that is, LiN(SO_2_CF_3_)_2_)—does not ameliorate this poor performance to any significant extent^12^. The search for other solvents for this application has also been problematic. Electrolytes based on ether solvents generally result in a less-dendritic Li morphology and improved CE for Li plating/stripping owing to their lower reactivity with Li metal^10^, but these features are not retained on prolonged cycling^5^. For example, Aurbach and Granot^15^ demonstrated that 1 M electrolytes with shorter chain glyme solvents (that is, 1,2-dimethoxyethane (DME or monoglyme), 1,2-diethoxyethane and diglyme) and various salts (that is, LiAsF_6_, LiBF_4_, LiClO_4_, LiCF_3_SO_3_, LiTFSI, LiI and LiBr) resulted in poor Li cycling efficiencies due to the formation of lithium alkoxy species (ROLi), which covered the Li surface. These glyme solvents were, however, noted to be less reactive than cyclic ethers, esters and alkyl carbonates.

Interestingly, Jeong *et al.*^16^ reported in 2008 that a CE of ~80% could be retained on cycling Li metal in a concentrated PC-based electrolyte with the LiBETI (that is, LiN(SO_2_C_2_F_5_)_2_) salt. Several years earlier, it was reported by Henderson (one of the coauthors in this work), as well as other researchers, that highly concentrated liquid electrolytes prepared with glyme solvents and lithium salts have both a relatively high ionic conductivity and high oxidative stability^17^,^18^,^19^,^20^,^21^,^22^. More recently, a number of publications have shown the striking differences between the properties and cell performances for dilute and highly concentrated electrolytes with glyme solvents, as well as other aprotic solvents^23^,^24^,^25^,^26^,^27^,^28^,^29^. One study, in particular, found that highly concentrated LiTFSI-1,3-dioxolane (DOL)/DME electrolytes inhibited Li metal dendritic growth, but the Li plating/stripping CE was reported to be rather low (~71%)^24^. Lu *et al.*^11^ reported that stable Li depositions can be obtained in the liquid electrolytes reinforced with halogenated salt blends with no signs of deposition instabilities over hundreds of cycles of charging and discharging. Zheng *et al.*^8^ used interconnected hollow carbon nanospheres to stabilize Li surface with a CE of ~99% for >150 cycles. However, no electrolytes have been reported to date that simultaneously overcome all of the challenges—dendritic metal deposition, low CE during Li deposition/stripping processes and poor rate performance—of Li metal electrodes. Most of the approaches reported to date mainly worked at relatively low current densities (<1 mA cm^−2^), which are insufficient to meet the requirements for many practical applications (>3 mA cm^−2^)^2^,^8^,^10^,^11^. Therefore, new electrolytes that can lead to the high rate and stable cycling of the Li metal anode are urgently needed for the further development of rechargeable Li metal batteries.

Here we demonstrate that the use of highly concentrated electrolytes composed of ether solvents and the salt lithium bis(fluorosulfonyl)imide (LiFSI or LiN(SO_2_F)_2_)^30^ results in the dendrite-free plating of Li metal at high rates and with high CE. This exceptional performance cannot be achieved when lower concentration electrolytes are used (with or without LiFSI) and when LiFSI is substituted with other salts. The fundamental mechanism behind the excellent performance of these electrolytes will also be discussed.

## Results

### Li metal deposition morphology

**Figure 1:**
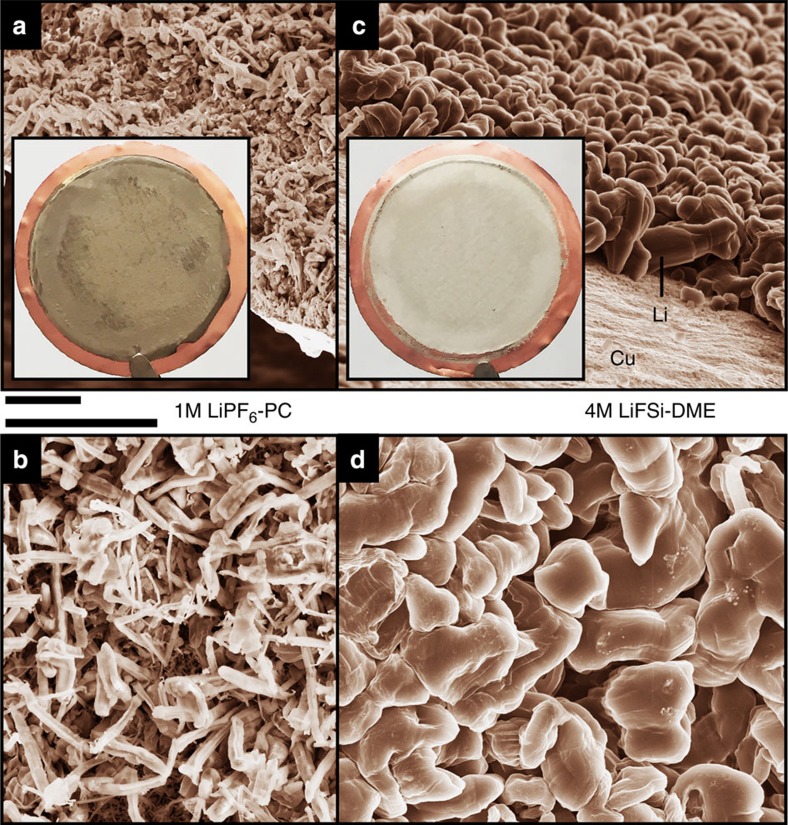
SEM images of the morphologies of Li metal after plating on Cu substrates in different electrolytes. (**a**,**b**) 1 M LiPF_6_-PC. (**c**,**d**) 4 M LiFSI-DME. The current density was 1.0 mA cm^−2^ and the deposition time was 1.5 h. The diameter of the Cu substrate shown in the insert of (**a**,**c**) was 2 cm. Scale bar, 10 μm.

The morphology of Li deposition in different electrolytes was evaluated by using coin-type Cu|Li cells (see Methods section for the details of the cell preparation). After the initial deposition, the coin cells were disassembled to collect the Li films deposited on the Cu substrates for microscopic analysis by scanning electron microscopy (SEM) without exposing to air. Figure 1a,b shows the cross-section and surface morphologies of the Li films deposited in a typical carbonate-based electrolyte (1 M LiPF_6_ in PC), respectively. In agreement with previous reports^9^,^12^, the plating of Li metal from a 1 M LiPF_6_-PC electrolyte resulted in extensive dendritic Li metal deposition. Similar dendritic Li growths were also obtained when other Li salts (such as LiTFSI and LiFSI) were used with PC as shown in Supplementary Fig. 1. In contrast, a nodule-like Li deposition without dendrite formation was obtained when Li film was deposited from the ether-based LiFSI-DME electrolytes with different salt concentrations (1–5 M) at various current densities (0.5, 1, 2 and 4 mA cm^−2^). Figure 1c,d shows the cross-section and surface morphologies of the Li film obtained in a concentrated 4M LiFSI-DME electrolyte. By comparing Fig. 1a,b (Supplementary Fig. 1) and Fig. 1c,d (Supplementary Fig. 2), two notable advantages can be identified in the morphologies obtained in the 4M LiFSI-DME electrolyte over those obtained in the carbonate-based electrolyte. First, the typical Li deposited in carbonate-based electrolyte exhibits a needle-like structure with a width of a few hundred nanometres, which can easily penetrate most conventional separators. In contrast, the typical Li deposited in the LiFSI-DME electrolytes exhibits a nodule-like structure with round-shaped edges. The typical dimension of the nodule-like Li particles is on the order of ~10 μm which restricts their ability to penetrate the porous separators. The second advantage is that the surface area of the films deposited in the ether-based electrolytes is much smaller than that obtained in carbonate-based electrolytes. Therefore, the side reactions between the deposited Li in the ether-based electrolytes will be much less than for Li deposited in carbonate-based electrolytes and will lead to higher CE during Li deposition/stripping processes. The optical images of the Li deposits in these two electrolytes also show a significant difference from each other. The inset of Fig. 1a shows the optical image of Li deposited on the Cu substrate (the diameter of the substrate is 2 cm). A typical dark grey Li deposit, often called Elton’s Grey Layer^31^, was observed for the electrode with the LiPF_6_-PC electrolyte (inset of Fig. 1a), indicating that the PC-based electrolyte is highly reactive with the plated Li to form a thick SEI layer on it. In contrast, the deposited Li from LiFSI-DME electrolytes is silver white shiny, close to the colour of pristine Li metal, indicating that the DME-based electrolytes are much more stable with Li metal.

### Li metal plating/stripping cycling stability

**Figure 2:**
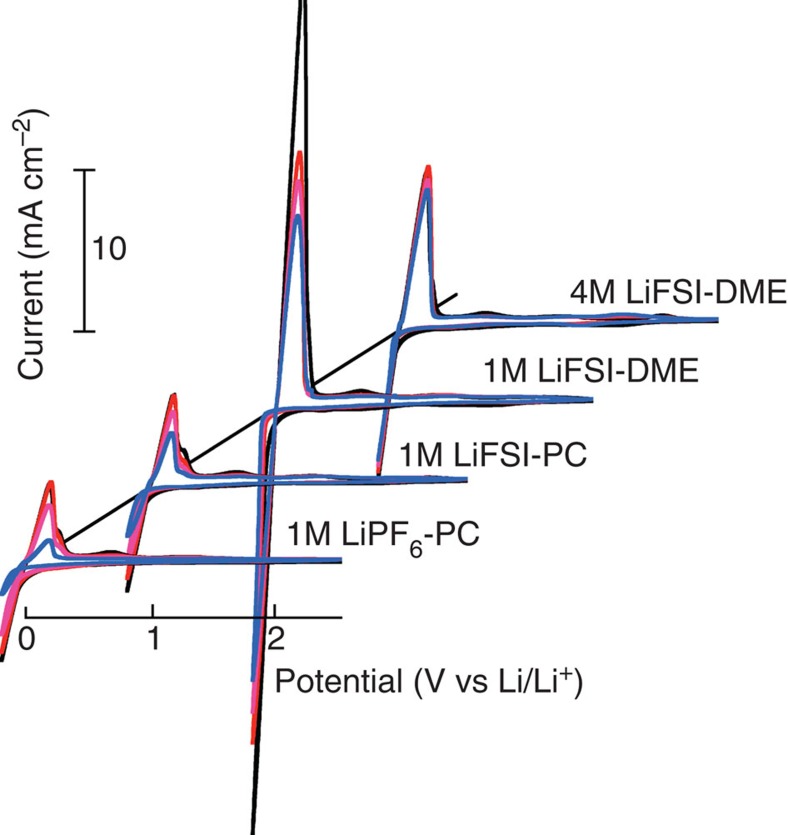
Cyclic voltammograms of Li plating/stripping in different electrolytes using a Pt disc (2 mm in diameter) as a working electrode and a Li metal as reference and counter electrode. The scan rate was 50 mV s^−1^.

Cyclic voltammetry (CV) was used to evaluate the Li plating (negative scan) and stripping (positive scan) behaviour in different electrolytes, where a Pt disk (2 mm in diameter) was used as the working electrode and Li metal as the reference and counter electrode as shown in Fig. 2. In general, the LiFSI-DME electrolytes—when cycled on Pt working electrodes—underwent negligible oxidation until a potential >4.5 V (Supplementary Fig. 3), suggesting that they may be used with a wide range of cathode materials. Notably, the PC-based electrolytes resulted in a relatively low-peak current density (~2 mA cm^−2^) for the plating/stripping and the current decreased substantially on cycling as shown. This may be due to the formation of a resistive SEI layer, which continued to grow on cycling^7^,^12^. In contrast, a much higher initial peak current density (~28 mA cm^−2^) was obtained for the cell tested in the 1M LiFSI-DME electrolyte. However, this current density declined rapidly on cycling, perhaps due to the reaction of the abundant solvent present with the plated Li metal. The current for the 4M LiFSI-DME electrolyte was lower by comparison, but this current intensity exhibits only minimal change on cycling possibly due to the lesser amount of un-coordinated solvent present in the electrolyte available to react with the freshly plated Li metal. It is also found that cells with either 2M LiFSI-DME electrolyte or 3M LiFSI-DME electrolyte exhibited more severe changes as compared with those of the 4 M LiFSI-DME in their CV profiles during repeated cycling (Supplementary Fig. 4). Although the CV curves obtained in the 5M LiFSI-DME electrolyte also exhibited a stable behaviour, a significant decrease in the conductivity of the 5 M LiFSI-DME (1.7 ms cm^−1^) as compared with that of the 4 M LiFSI-DME (5.7 ms cm^−1^, see Supplementary Table 1) rendered this electrolyte less favourable for the high-rate cycling of Li metal electrode. These results indicated that 4 M LiFSI in DME is an optimized salt concentration to obtain the stable cycling of Li metal in this electrolyte system.

**Figure 3:**
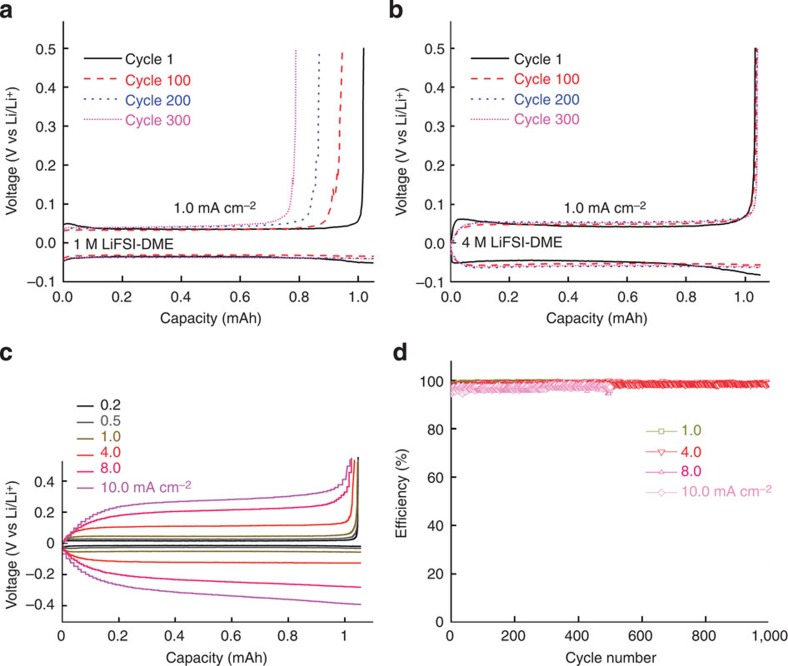
Electrochemical performance of Li metal plating/stripping on a Cu working electrode. (**a**) Voltage profiles for the cell cycled in 1 M LiFSI-DME; (**b**) Voltage profiles for the cell cycled in 4 M LiFSI-DME; (**c**) Polarization of the plating/stripping for the 4 M LiFSI-DME electrolyte with different current densities. (**d**) CE of Li deposition/striping in 4 M LiFSI-DME at different current densities.

The coin-type Cu|Li cells with different electrolytes were used to investigate the cycling stability of Li plating/stripping in this work. The CE of the Li plating/stripping can be calculated from the ratio of Li removed from Cu substrate to that deposited during the same cycle (as reflected by the total charge for each process). Significant differences in the voltage profiles of the Cu|Li cells with different electrolytes were observed. Figure 3a,b compares these voltage profiles at different stages of the cycling using 1 and 4M LiFSI-DME electrolytes, respectively. For the 1 M LiFSI-DME cell cycled at 1.0 mA cm^−2^, although the charging behaviour remained the same over 300 cycles, less and less Li could be stripped from the Cu electrode as the cycle number increased. This means that a significant amount of the Li deposited on the substrate reacted with the electrolyte and could not be recovered during the stripping process. However, the voltage profile of Cu|Li cell with the highly concentrated 4M LiFSI-DME electrolyte is highly stable for >300 cycles (Fig. 3b) during both the charge and discharge processes. Remarkably, although the polarization of the cells increases with increasing current density (Fig. 3c), Cu|Li cells with this electrolyte could be stably cycled at a high rate of 4.0 mA cm^−2^ for >1,000 cycles with a stable CE of 98.4%, as shown in Fig. 3d. Even at a very high rate of 10.0 mA cm^−2^, a stable CE of >97% was maintained for >500 cycles. The average CE values over 500 Li plating/stripping cycles at current densities of 0.2 and 1.0 mA cm^−2^ are 99.1 and 98.5%, respectively. In sharp contrast, although a nodule-like structure was also observed for Li deposited in 1 M LiFSI-DME (Supplementary Fig. 1), the CE in this electrolyte exhibits large variation and fades quickly after 100 cycles (Supplementary Fig. 5). Importantly, the cycling behaviour of the electrolytes improves for the more concentrated LiFSI-DME electrolytes, especially for high current densities, despite the fact that the ionic conductivity decreases with increasing salt concentration (Supplementary Table 1). It is noted that a nodule-like shape structure was also observed when Li was deposited in a 1-M LiFSI/LiTFSI-DOL/DME electrolyte (1/1 salt mole ratio and 2/1 solvent volume ratio)^32^ or 1-M LiAsF_6_-DOL electrolyte stabilized with tributylamine^4^. However, the long-term cycling profiles and the CEs of Li plating/stripping in these electrolytes are far less stable than those observed in highly concentrated (4 M) LiFSI-DME electrolyte.

**Figure 4:**
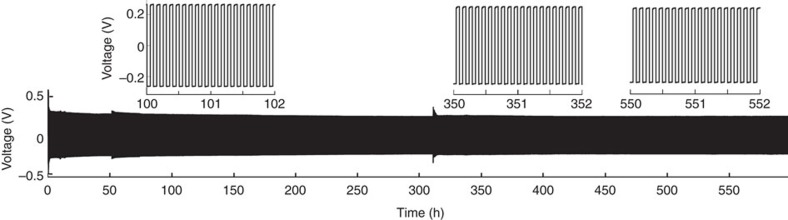
Li metal plating/stripping from a Li|Li cell cycled at 10.0 mA cm^−2^ with a 4 M LiFSI-DME electrolyte. The top plots are expanded views from the bottom plot.

A symmetric Li|Li cell was used to further investigate the stability of the Li metal anode in the concentrated electrolyte. Figure 4 shows the long-term cycling stability of a coin-type Li|Li cell with the 4M LiFSI-DME electrolyte. An exceptionally high current density of 10.0 mA cm^−2^ was used and the cell was cycled for over 600 h, which corresponds to 6,000 charging/discharging cycles (cycling capacity was 0.5 mA cm^−2^ for both charging and discharging processes). These results further demonstrate the exceptional cycling stability of the highly concentrated 4M LiFSI-DME electrolyte, which enables the reversible plating/stripping of Li metal in a Li|Li cell at a high rate for many thousands of cycles. In contrast, the cycling of Li|Li cells with the 1M LiTFSI-DME electrolyte at this and lower current densities often results in increased voltage polarizations and random voltage oscillations, as shown in Supplementary Fig. 6. The final result of this behaviour is either a short circuit due to the dendrite growth or the high-impedance-related cell failure.

### Formation of a stable SEI layer

**Figure 5:**
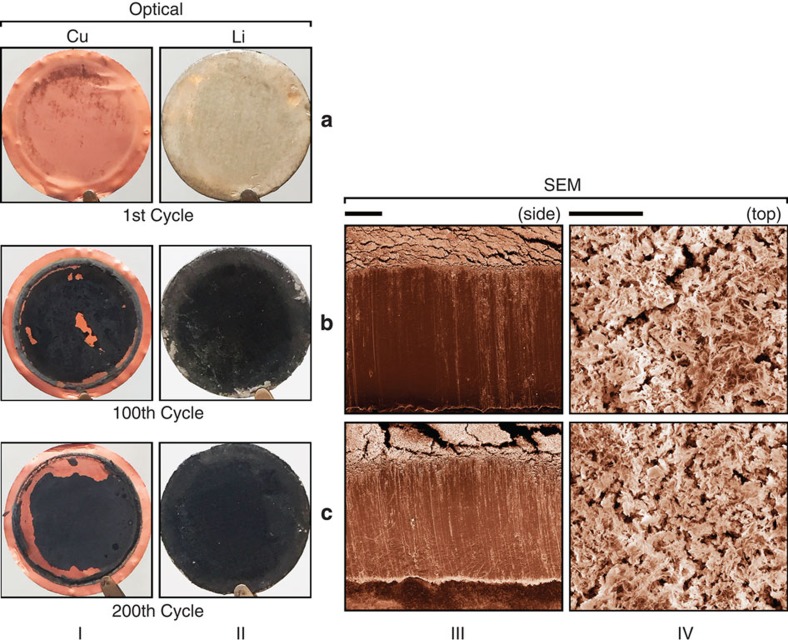
Optical and SEM images of the cycled Cu and Li electrodes. Electrodes were harvested from Cu|Li cells cycled at 4.0 mA cm^−2^ with a 4-M LiFSI-DME electrolyte after: (**a**) 1st cycle. (**b**) 100th cycle. (**c**) 200th cycle. Columns I and II are, respectively, for optical images of Cu and Li electrodes at different cycle numbers, while Columns III and IV show the SEM images of the cross-section and surface morphologies of Li metal anode. The diameter of the Cu substrate shown in Column I and II was 2 cm. The scale bars for Columns III and IV are 100 and 10 μm, respectively.

To identify the mechanism behind the long-term cycling stability of Li metal electrode in the highly concentrated LiFSI-DME electrolytes, the morphology evolution of the SEI layer formed on the Li surface was investigated by SEM and optical images. In the literature, several groups have reported the corrosion of Li metal electrode as another failure mechanism of Li metal electrode in addition to dendrite growth^7^,^33^,^34^. In contrast, a largely different morphology was observed for the Li metal electrode cycled in the 4M LiFSI-DME electrolyte. In this case, a relatively compact SEI layer was formed on the surface of Li metal anode as shown in Fig. 5, but no porous Li metal was observed as was found in other electrolytes. Figure 5a shows the optical images of the Cu and Li electrodes after the first plating/stripping cycle. Only a light residual layer was observed after the Li was removed from the Cu substrate. The optical images of the Cu (column I) and Li (column II) electrodes after the 100th and 200th cycles are shown in Fig. 5b,c, respectively. A black SEI layer was formed on both the Cu and Li surfaces after extensive cycling. The thickness of the black SEI layer grows slowly (~10 μm after 100 cycles). This is consistent with a CE of 98% (Fig. 3d), indicating that a small amount of charge has been consumed by side reactions, which produce the dark SEI layer evident in Fig. 5b,c. The SEM images of the cross-section and surface of the Li electrode were shown in columns III and IV of Fig. 5, respectively. The cross-sectional view of the cycled Li metal showed that the black SEI layer was compact and only formed on the Li surface. Although some cracks on the surface of the Li metal electrode were introduced during the SEM sample preparation process, there is no identifiable corrosion observed inside of the Li electrode even after 200 cycles. This means that the SEI layer formed on the Li electrode surface is highly compact and can prevent further corrosion of Li electrode, which is critical for achieving a high CE and long-term cycling of Li metal electrode. More importantly, in contrast to the highly resistive nature of the thick SEI formed when Li metal is cycled in other electrolytes^7^,^33^, the interfacial resistance (which is dominated by the impedance of the SEI layer) of the plated Li metal remains low when Li metal is stored in the 4M LiFSI-DME electrolyte compared with those stored in 1M LiPF_6_-PC and 1M LiFSI-DME (Supplementary Fig. 7) for 48 h. Surprisingly, the impedance of the Cu|Li cell decreases with increasing cycle number (Supplementary Fig. 8) when cycled in 4M LiFSI-DME electrolyte. This is in sharp contrast to those observed for the behaviour of Cu|Li cells with other electrolytes. These results indicate that the black SEI layer formed on the surface of the Li (or Cu) electrode (see Fig. 5) is not only highly compact (which prevents further corrosion of Li metal below the SEI layer), but also highly conductive, so that the cell voltage does not increase even after 6,000 cycles at very high current densities (10 mA cm^−2^), as shown in Fig. 4.

Furthermore, X-ray photoelectron spectroscopy (XPS) analysis was used to analyse the compositions of the SEI layer formed on the Li surface plated on a Cu electrode using the 4M LiFSI-DME electrolyte (Supplementary Fig. 9 and Supplementary Table 2). Notably, the surface layer after the initial plating of the Li metal contains negligible amount of anion components. The increase of the sulfur, fluoride and nitrogen contents on cycling may be due to anion degradations. Supplementary Table 2 also shows that composition of the SEI layer is relatively stable after initial cycles. The build-up of the SEI layer (Fig. 5) for the 4M LiFSI-DME electrolyte appears to be linked with slow anion degradation during extended cycling, which results in the surface layer having a significant amount of inorganic components.

## Discussion

**Figure 6:**
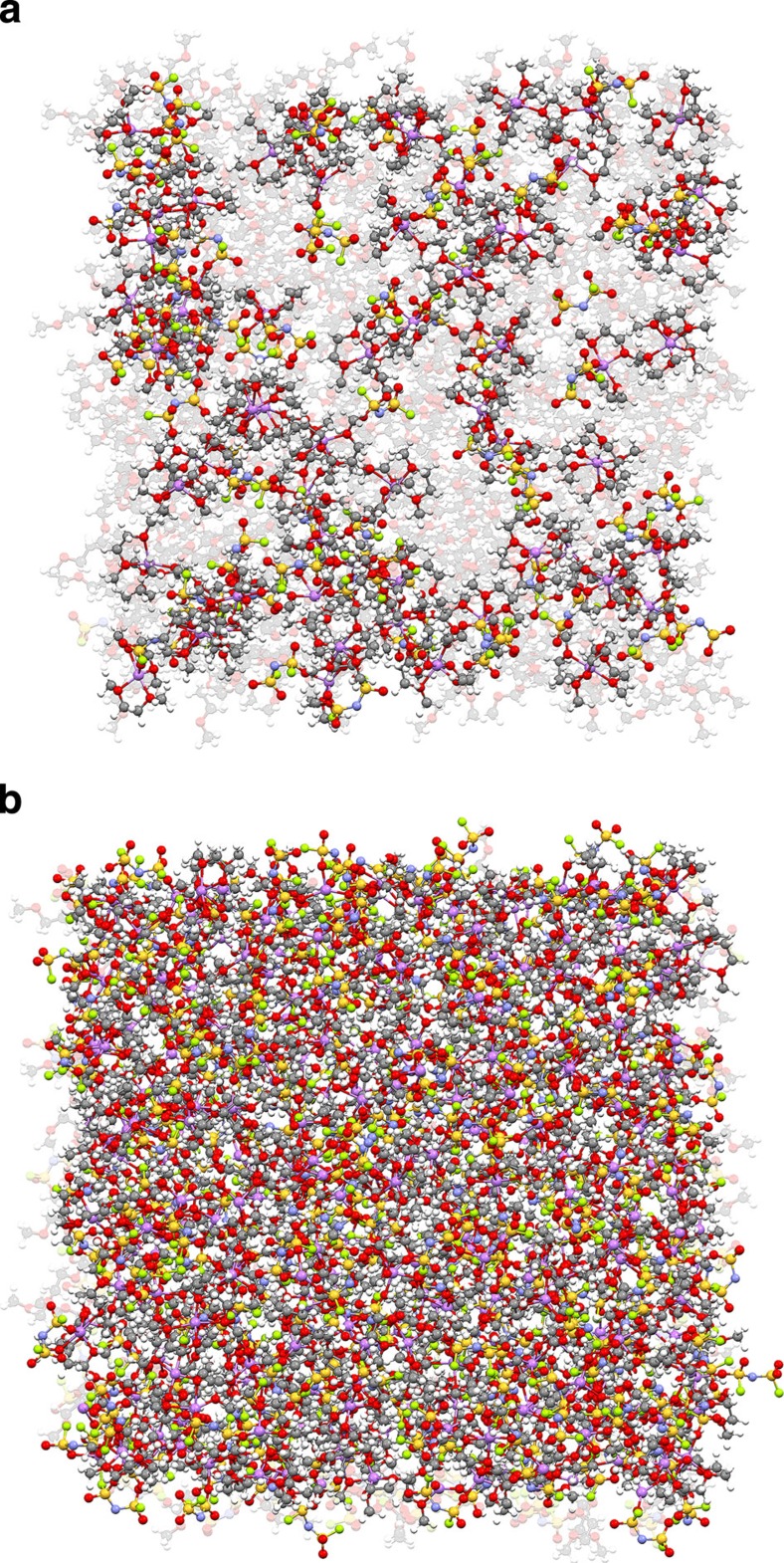
Snapshots of the MD simulation boxes. (**a**) A 1- M LiFSI-DME electrolyte, (**b**) 4-M LiFSI-DME electrolyte. Colours for different elements: Li-purple, O-red, N-blue, S-yellow and F-green. The uncoordinated DME solvent molecules are coloured light grey.

Three key factors contributed to the excellent high-rate cycling stability of Li metal anode in the high concentration LiFSI-DME electrolyte. The first factor is the electrolyte solvent. DME was reported to have the lowest reduction potential of −1.68 V against Li/Li^+^ among various linear ethers, implying that the probability of a direct reaction between DME solvent and Li metal would be low^10^. It also demonstrates the best stability during Li deposition among all Li|Li symmetric cells using different 1-M LiTFSI-linear ether electrolytes, indicating that DME is the solvent that is most effective in reducing dendritic growth^10^. Another factor is the selection of the electrolyte salt. LiFSI results in electrolytes with a high ionic conductivity due to the weak interaction between the solvated Li^+^ cations and FSI^−^ anions^35^,^36^. As compared with LiPF_6_-carbonate solvent electrolytes, those with LiFSI have a higher ionic conductivity over a wide temperature range (−50 to 50 °C) and a higher Li^+^ transference number (*t*_Li+_) of 0.5–0.6 (ref. 35). In addition, this salt has a high solubility in most polar solvents and low tendency to crystallize as high-melting solvate phases, resulting in liquid electrolytes at ambient temperature even for extremely high salt concentrations. However, these two factors are not enough to ensure the high-rate cycling stability of Li metal anode as demonstrated by the relatively poor cycling performance of the 1-M LiFSI-DME electrolyte. To aid in understanding the differences in the performance of the dilute (1 M) and highly concentrated (4 M) LiFSI-DME electrolytes, molecular dynamic (MD) simulations were performed to examine the solution structure of the bulk electrolytes. These electrolytes correspond to ~9/1 and 1.4/1 mole ratios of DME/LiFSI (Supplementary Table 3). Figure 6 shows snapshots of the simulation boxes (that is, the simulation at a fixed time during the equilibrium run period) for the 1- and 4-M LiFSI-DME electrolytes with the periodic boundaries unwrapped. The uncoordinated DME solvent is shown as a lighter, faded colour to assist in viewing the solvates and uncoordinated anions. Examples of the distribution/population of the uncoordinated FSI^−^ anions and various solvate species extracted from such snapshots are shown in Supplementary Figs 10 and 11. For the 1-M electrolyte, a large fraction of the solvent molecules are uncoordinated. Approximately 60% of the FSI^−^ anions and Li^+^ cations are uncoordinated and fully solvated (Supplementary Table 3), respectively, while the majority of the remainder of the ions are present as solvated ion pairs. The coordination interactions within the 4-M electrolyte, however, are markedly different. Only about 3% of the anions are uncoordinated and 6% or so of the Li^+^ cations are fully solvated. Nearly all of the ions are instead present as contact ion pairs and aggregate solvates (both as small and large to very large aggregate clusters).

These results explain the much higher viscosity noted for the 4-M LiFSI-DME electrolyte relative to the 1-M electrolyte, as well as the decrease in the ionic conductivity (Supplementary Table 1, from 16.9 ms cm^−1^ for 1 M to 5.7 ms cm^−1^ for 4 M). Why then should the cells with the highly concentrated electrolyte perform far superior to the more dilute electrolytes in terms of rate capability and stability (Fig. 3 and Supplementary Fig. 5)? This can be attributed principally to the following two factors:

1. improved electrolyte reductive stability due to reduced availability of reactive solvent and sacrificial anion reduction;
2. increased Li^+^ concentration enables high-rate Li plating/stripping.

Battery electrolyte concentrations of ~1 M are widely utilized because the ionic conductivity of the electrolytes (for diverse aprotic solvents and Li salts) tends to peak at this concentration. It is often assumed that maximizing the electrolyte conductivity results in an optimal battery rate (power) capability. It was recently demonstrated for Li|graphite cells, however, that the use of highly concentrated electrolytes with either LiTFSI or LiFSI and acetonitrile or DME resulted in a far superior high rate performance than that for the state-of-the-art electrolytes (for example, 1 M LiPF_6_-EC/DMC) used in commercial Li-ion batteries^26^,^27^.

During Li plating, the charge density on the negatively polarized Li metal surface provides electrons that can reduce neighbouring Li^+^ cations or may instead react with other electrolyte components (that is, solvent molecules and/or anions). The real reaction route is related to the availability of the Li^+^ cations and the reactivity of the other species. The polarized Li metal interface will create an electric double-layer, but this can be expected to include a considerable amount of solvent for a 1M electrolyte^37^. Carbonate solvents rapidly react with such a polarized interface. The more stable ether solvents (for example, DME) are slower to react, but still may do so. This slower reactivity provides more time for the Li^+^ cations to migrate in the electric field to the electrode surface, but plating at high current density may deplete the amount of Li^+^ cations available for reduction at the electrolyte/electrode interface. Although the 4M electrolyte has a lower conductivity than the 1M electrolyte, there are four times as many Li^+^ cations within a given volume for the former electrolyte. Thus, the proximity of the Li^+^ cations is much closer (Fig. 6 and Supplementary Fig. 12) and the distance necessary for Li^+^ cation transport to maintain a given Li^+^ cation flux is therefore much shorter. In addition, the flux is a function of both conductivity and diffusion (as well as convection, if the electrolyte flows)^38^. For Li plating at high current density, as the Li^+^ cations near the electrode surface react and become depleted, diffusion (due to the resulting concentration gradient) will contribute to the Li^+^ cation flux to a greater extent for the highly concentrated electrolyte (relative to the more dilute electrolyte). Figure 6 also shows that a significant fraction of the volume is due to the anions of the 4-M electrolyte. Anions, in general, are less prone to reduction (than aprotic solvent molecules) that may further stabilize the charge density on the Li metal surface sufficiently for the Li^+^ cation flux to accommodate the transport and subsequent reduction of the Li^+^ cations, thereby resulting in the high CE reported during extensive cycling, even for Li plating/stripping at high current densities (Fig. 3c). When the anions do react with the Li metal (anions coordinated to Li^+^ cations are expected to be more susceptible to reduction than uncoordinated anions, known as sacrificial anion reduction mechanism)^32^,^39^, rather than producing a highly resistive layer comprised principally of ROLi species from the reduction of ether solvents, the SEI layer may instead have a greater amount of inorganic components (than for a 1-M electrolyte), which results in a low resistance to Li^+^ cation transport (Supplementary Figs 7 and 8).

In conclusion, compact and non-dendritic Li metal can be plated/stripped from a Cu electrode at high rates with a high Coulombic efficiency by using concentrated electrolytes based on ether solvents and the LiFSI salt. The reactivity of these electrolytes is low (resulting in very limited side reactions and thus a high CE) and the large amount of Li^+^ cations available enables high current densities to be used for Li metal deposition. For more dilute electrolytes, the solvent is found to react with the plated Li metal to a much greater extent, which lowers the CE of the Li plating/stripping. Although the thickness of the SEI layer still grows with increasing cycle numbers as a result of the non-perfect CE (~99%) for Li plating/stripping in the 4M LiFSI-DME electrolyte, the highly conductive nature of the SEI layer leads to a highly stable voltage profiles during the cycling of Li electrode. In addition, the highly compact feature of the SEI layer also prevents further corrosion of the Li metal electrode and results in excellent stability of the electrode in the highly concentrated LiFSI-DME electrolytes. The Li plating/stripping performance of the highly concentrated LiFSI-ether electrolytes, therefore, far exceeds that of standard concentration (that is, 1 M) electrolytes. It is demonstrated that a Cu|Li cell can be cycled at 4 mA cm^−2^ for >1,000 cycles with a high CE of 98.4% in the 4-M LiFSI-DME electrolytes. This study provides a route for future efforts to optimize electrolytes for the safe and highly efficient utilization of Li metal electrodes for advanced energy storage applications.

## Methods

### Materials

Lithium hexafluorophosphate (LiPF_6_), lithium bis(trifluoromethanesulfonyl)imide (LiTFSI), 1,2-dimethoxyethane (DME), 1,3-dioxolane (DOL) and propylene carbonate (PC) (all in battery-grade purity) were obtained from BASF Corporation. Lithium bis(fluorosulfonyl)imide (LiFSI) was obtained from Nippon Shokubai and used as-received. The electrolytes were prepared by dissolving the desired amount of salt into the solvent. Li foil and Cu foil were purchased from MTI Corporation and All Foils, respectively. The materials were stored and handled in an MBraun LABmaster glove box with an Ar atmosphere (<1 p.p.m. O_2_ and <1 p.p.m. H_2_O).

### Electrochemical measurements

Cyclic voltammetric studies of the electrolyte solutions were conducted in a three-electrode configuration inside the glove box using a CHI606E workstation. The working electrode was a 2.0 mm diameter Pt disk (from CH Instrument) and Li metal was used as both the counter and the reference electrodes. Electrochemical cycling tests were carried out using CR2032-type coin cells of a two-electrode configuration. Coin cells (Cu|Li and Li|Li) were assembled in the glove box with Li foil used as both the counter and reference electrode. Celgard 2400 (polypropylene membrane) was used as the separator, while Cu foil served as the substrate for Li metal deposition. The Cu foil was washed by immersing it in 1 M HCl for 10 min, followed by rinsing separately with deionized water and acetone three times. The Cu foil was then quickly dried in a vacuum oven at room temperature. To standardize the testing, 75 μl of electrolyte was used in each coin cell. The current density for the Li metal plating/stripping was set to 0.2, 0.5, 1.0, 2.0, 4.0, 8.0 or 10.0 mA cm^−2^ using a Lanhe battery testing station at room temperature. The effective area of the Cu foil for Li deposition was 2.11 cm^2^ (diameter 1.64 cm). During each cycle, 0.5 mAh cm^−2^ of Li metal was deposited on the Cu substrate at various current densities and then stripped until the potential reached 0.5 V vs Li/Li^+^. Li|Li symmetric cells were assembled with Li metal used as the working and counter electrodes. Conductivity measurements were carried out using a Metrohm 644 conductometer with a cell made of two parallel Pt wires. The batteries were tested in a Tenney JR environmental chamber to ensure the stable temperature during long-term cycling process.

### Characterization

Cycled coin cells were dissembled to harvest Li metal discs and Cu substrates for characterizations of SEM and XPS. Before analysis, these electrodes were first rinsed with DME solvent three times to remove residual electrolytes and then dried in the vacuum chamber of the MBraun glove box. SEM images of the Li electrodes for both the surface and the cross-sections were obtained with an FEI Quanta FESEM at an accelerating voltage of 5 kV. A cross-section of Li metal was prepared by vertically cutting the Li samples with a razor blade. XPS measurements were performed on a Physical Electronics Quantera Scanning X-ray Microprobe. This system uses a focused monochromatic Al Kα X-ray (1,486.7 eV) source for excitation and a spherical section analyser. A 100 W X-ray beam focused to a 100 μm diameter was rastered over a 1.4 × 0.1 mm^2^ rectangular portion of the sample. The X-ray beam was incident normal to the sample and the photoelectron detector was 45° off-normal. High-energy-resolution (narrow scan) XPS spectra were collected using a pass-energy of 69.0 eV with a step size of 0.125 eV. All of the spectra were charge referenced using the C1s line at 285.0 eV for comparison purposes. To avoid electrode contamination or side reactions with atmospheric moisture and oxygen, the samples were transferred from the glove box to the SEM and XPS in sealed vessels, which were filled with Ar gas.

### MD simulations

Molecular dynamic (MD) simulations were performed at 333 K for two salt concentrations of LiFSI-DME mixtures: 0.96 and 4.24 M salt concentrations correspond to DME/LiFSI molar ratios of 9/1 and 1.4/1, respectively. The simulation cells contain 576 DME/64 LiFSI for the 0.96 M concentration and 448 DME/320 LiFSI for the 4.24 M salt concentration (Supplementary Table 3), respectively. The initial simulation box size of 80 Å reduced to about 50 Å (Supplementary Table 3) during 0.2 ns simulations at 450 K, followed by a 6 ns equilibration run at 363 K that was performed in an NPT ensemble. Following this, the MD simulations were run for 8 ns at 333 K in an NPT ensemble at *P*=1 atm. These runs were not included in the analysis and were considered to be equilibration runs. Production runs were performed at 333 K in the NVT ensemble using the average simulation box size from the NPT ensemble for 30 and 20 ns for the 0.96 and 4.24 M concentrations, respectively. A many-body polarizable force field (FF) APPLE&P (Atomistic Polarizable Potential for Liquids, Electrolytes, and Polymers) was used^40^. The LiFSI force field was largely taken from the recent works^41^,^42^,^43^ with the exception of the FSI^−^ anion oxygen polarizability, which was changed to 1.2 Å^3^ (cubic angstrom) to better reproduce the binding energies of LiFSI clusters obtained from quantum chemistry calculations at the G4MP2 level^42^. The DME force field parameters were taken from the previous work^40^ with the exception of the oxygen polarizability, which was set to 1.14 Å^3^ to match the DME(tgt)/Li^+^ binding energy of −59.4 kcal mol^−1^ obtained from G4MP2 quantum chemistry calculations. A detailed discussion of the functional form and simulation parameters is provided elsewhere^40^,^41^,^42^.

## Author contributions

J.-G.Z. and J.Q conceived and designed the experiments with the help from W.X. and W.A.H. J.Q. prepared the samples and performed the electrochemical measurements. P.B., M.E. and O.B. performed the SEM measurements, XPS measurements and MD simulations, respectively. W.A.H. proposed the explanations for the high rate capability of high-concentration electrolytes. W.A.H., J.Q., W.X. and J.-G.Z. prepared the manuscript.

## Additional information

**How to cite this article:** Qian, J. *et al.* High rate and stable cycling of lithium metal anode. *Nat. Commun.* 6:6362 doi: 10.1038/ncomms7362 (2015).

## Supplementary Material

Supplementary Information Supplementary Figures 1-12 and Supplementary Tables 1-3
