# Supplementary material for: High rate and stable cycling of lithium metal anode
Source: Nat Commun. 2015 Feb 20;6:6362. doi: 10.1038/ncomms7362 (PMC4346622; doi:10.1038/ncomms7362)
Supplement: Supplementary Information — Supplementary Figures 1-12 and Supplementary Tables 1-3 [file ncomms7362-s1.pdf]

Supplementary Information

Supplementary Figures

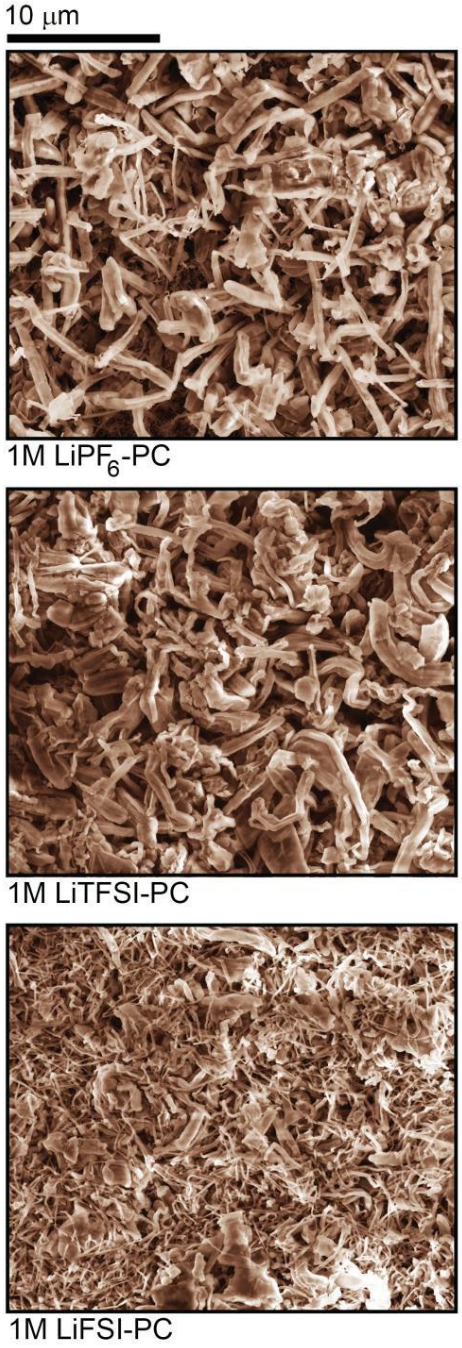

Supplementary Figure 1 | SEM images of the morphologies of Li metal after plating on Cu (1st cycle) from different electrolytes. The current density was 0.5 mA cm^{-2}.

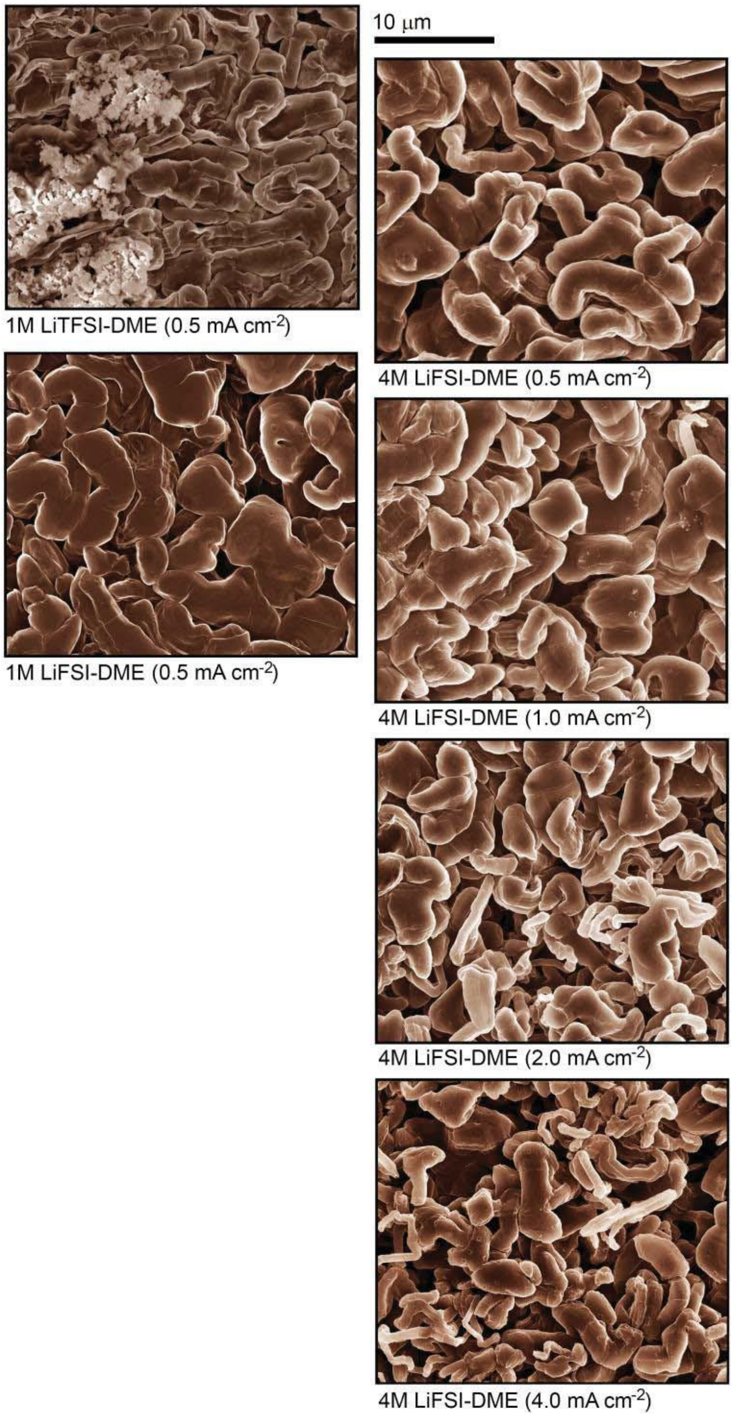

Supplementary Figure 2 | SEM images of the morphologies of plated Li metal after plating on Cu (1st cycle) from different electrolytes at different current densities.

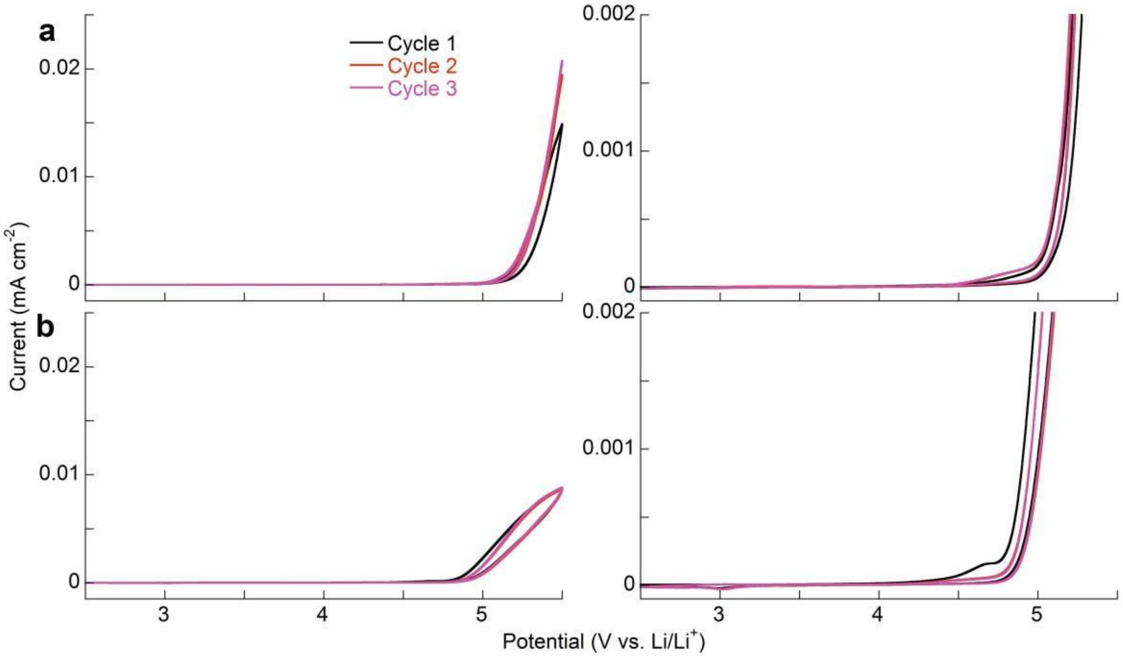

Supplementary Figure 3 | Cyclic voltammograms on Pt with the LiFSI-DME electrolytes. (**a**) 1 M LiFSI-DME, (**b**) 4 M LiFSI-DME. The plots on the right are scaled data from the plots on the left.

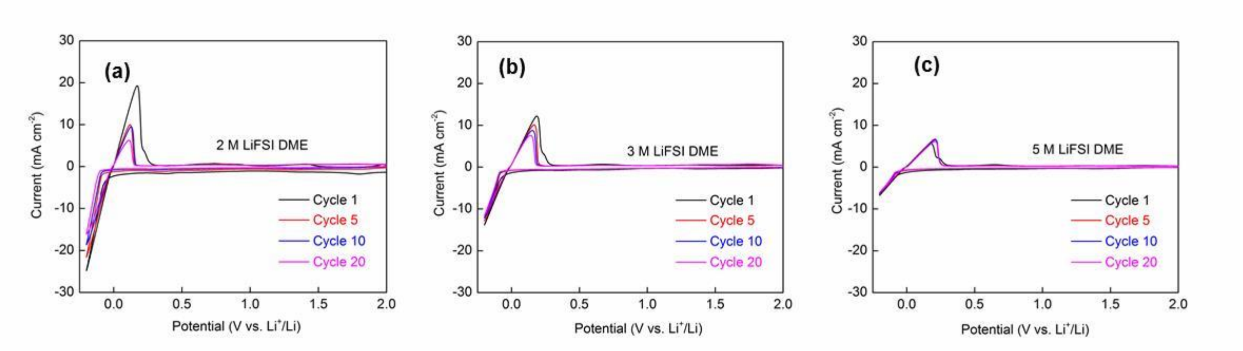

Supplementary Figure 4 | Cyclic voltammograms of Li|Pt cells in different electrolytes. The scan rate was 50 mV/s. (**a**) 2 M LiTFSI-DME, (**b**) 3 M LiTFSI-DME, and (**c**) 3 M LiTFSI-DME.

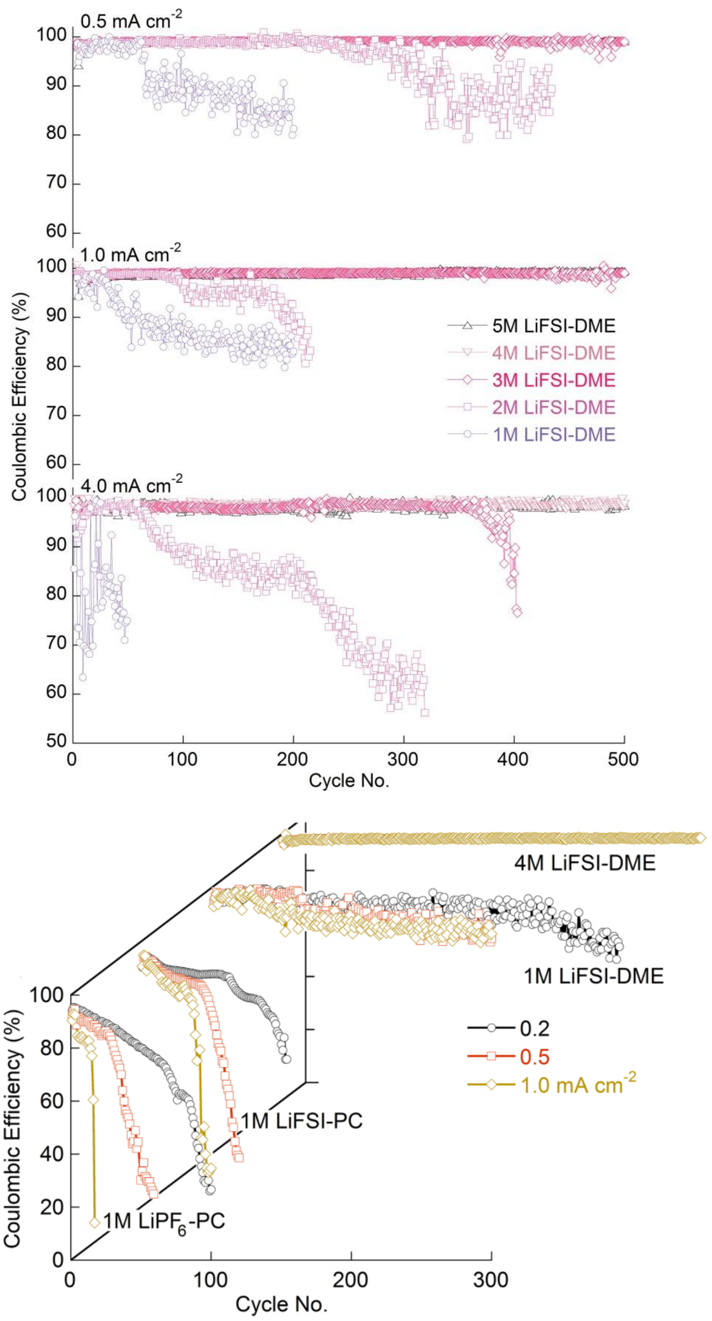

Supplementary Figure 5| Coulombic efficiency of Li|Cu cells cycled in LiFSI-DME electrolytes with different salt concentrations, 1 M $LiPF_6$-PC and 1 M LiFSI-PC at different current densities.

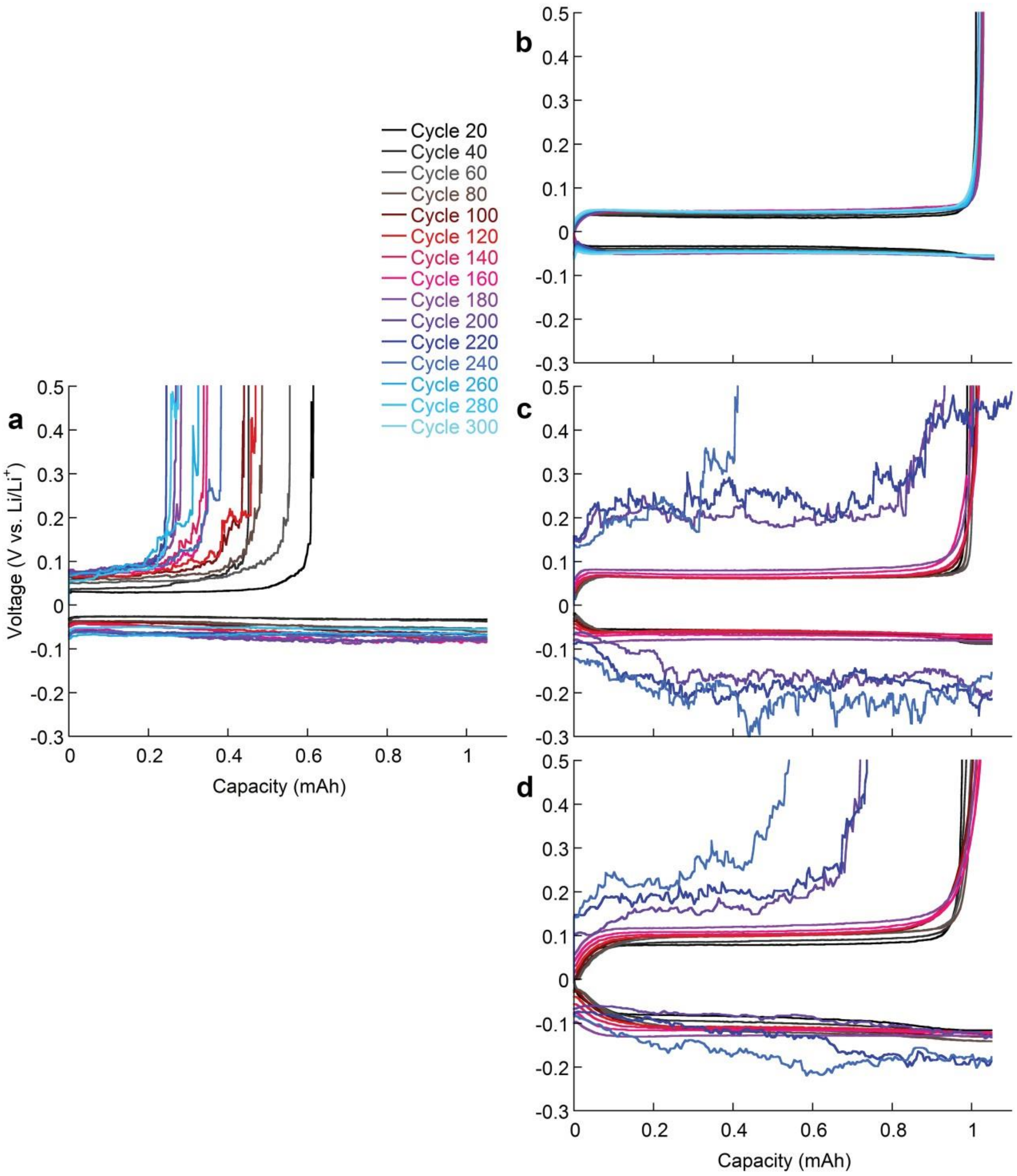

Supplementary Figure 6 | Li metal plating/stripping on Cu from LiTFSI-DME electrolytes at different current densities. (**a**) 1 M LiTFSI-DME at 0.5 mA cm^{-2}. (**b**) 3 M LiTFSI-DME at 0.2 mA cm^{-2}. (**c**) 3 M LiTFSI-DME at 0.5 mA cm^{-2}. (**d**) 3 M LiTFSI-DME at 1.0 mA cm^{-2}.

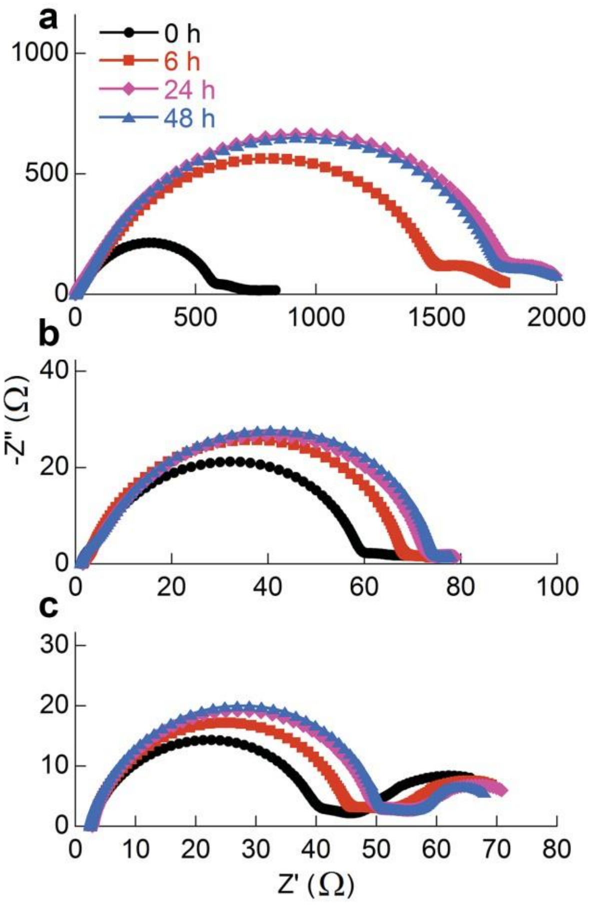

Supplementary Figure 7 | Impedance spectra of Li|Li cells with different electrolytes upon standing for the indicated periods of time. (**a**) 1 M $LiPF_6$-PC. (**b**) 1 M LiFSI-DME. (**c**) 4 M LiFSI-DME. The resistance of the cell does not change significantly for the 1 M and 4 M LiFSI-DME when these electrolytes are exposed to Li metal (i.e., when the Li metal surface is un-polarized the anion and DME solvent are relatively unreactive).

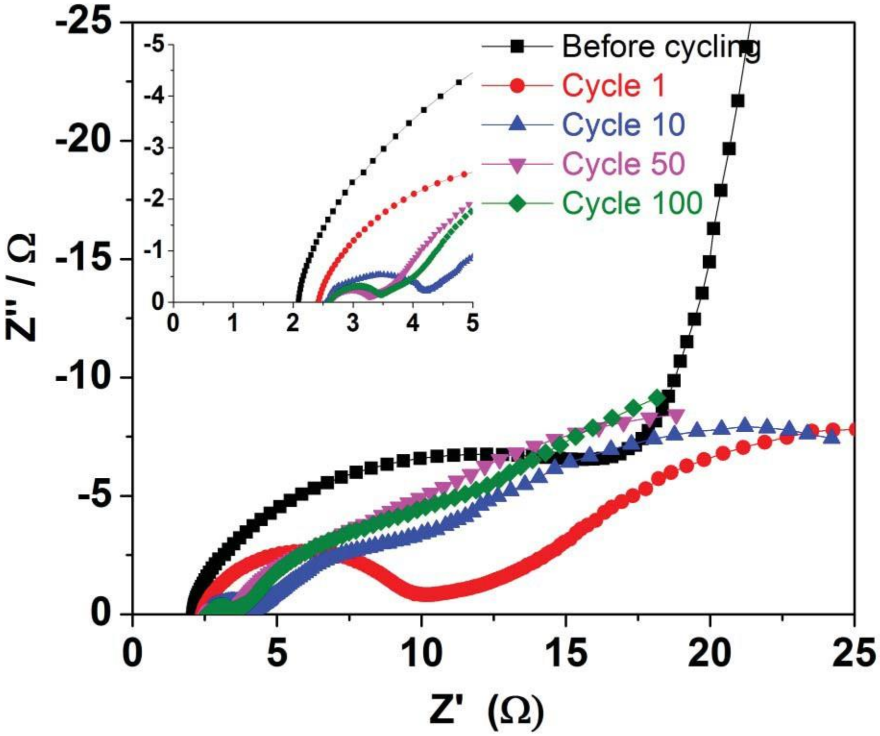

Supplementary Figure 8 | Impedance spectra of a Li|Cu cell with a 4 M LiFSI-DME electrolyte. The spectra were obtained prior to cell cycling and after the indicated cycles at a current density of 1 mA cm^{-2}. The impedance for a coin-type Li|Cu cell with a 4 M LiFSI-DME electrolyte actually decreases upon cycling. This is not the case, however, for the Li|Li cell with the 1M $LiPF_6$-PC electrolyte which formed a highly resistive interface with the Li metal.

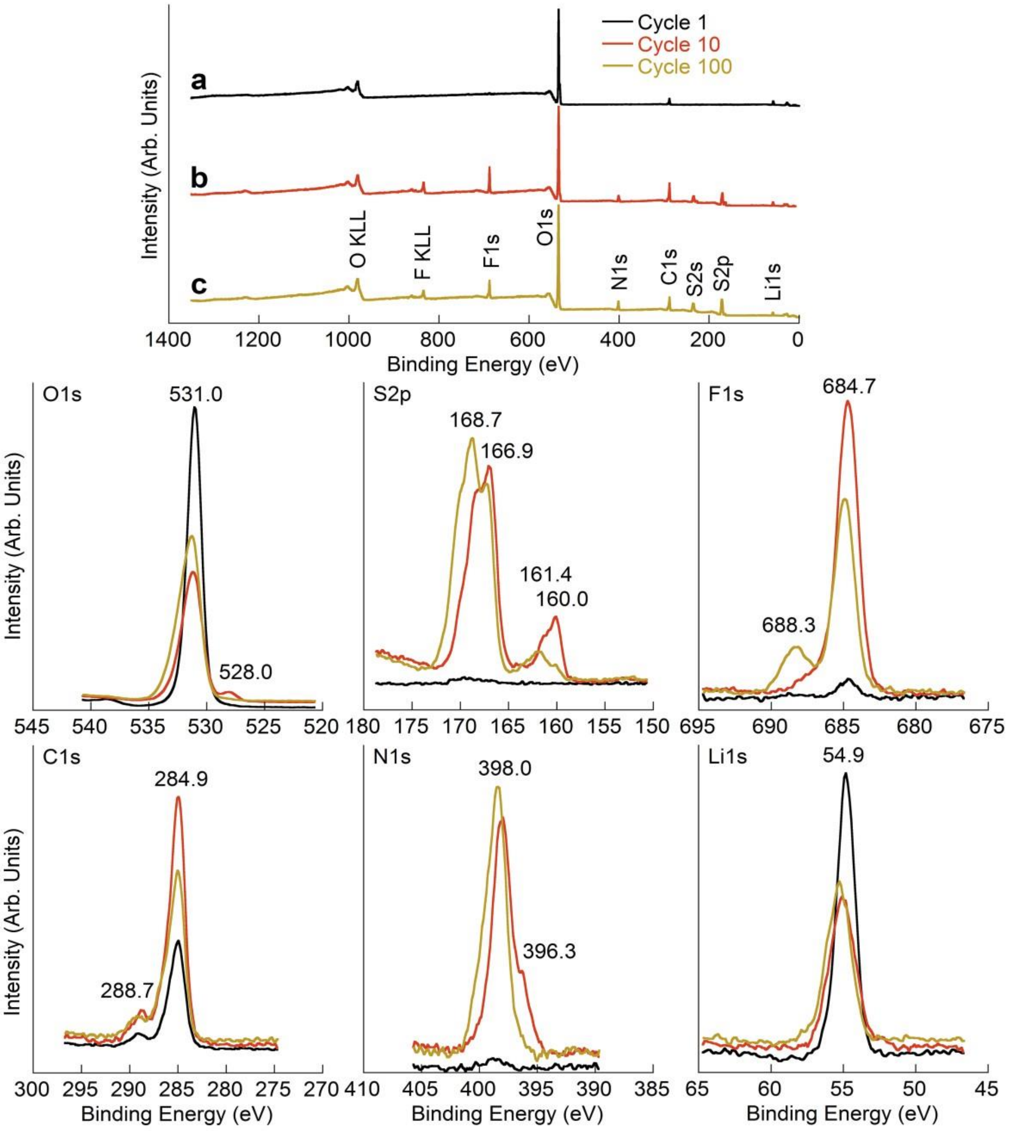

Supplementary Figure 9| XPS data for the Li surface plated on a Cu electrode using a 4 M LiFSI-DME electrolyte and a current density of 0.5 mA cm^{-2}. (**a**) Li deposited during Cycle 1 (black). (**b**) Li deposited during Cycle 10 (red). (**c**) Li deposited during Cycle 100 (tan).

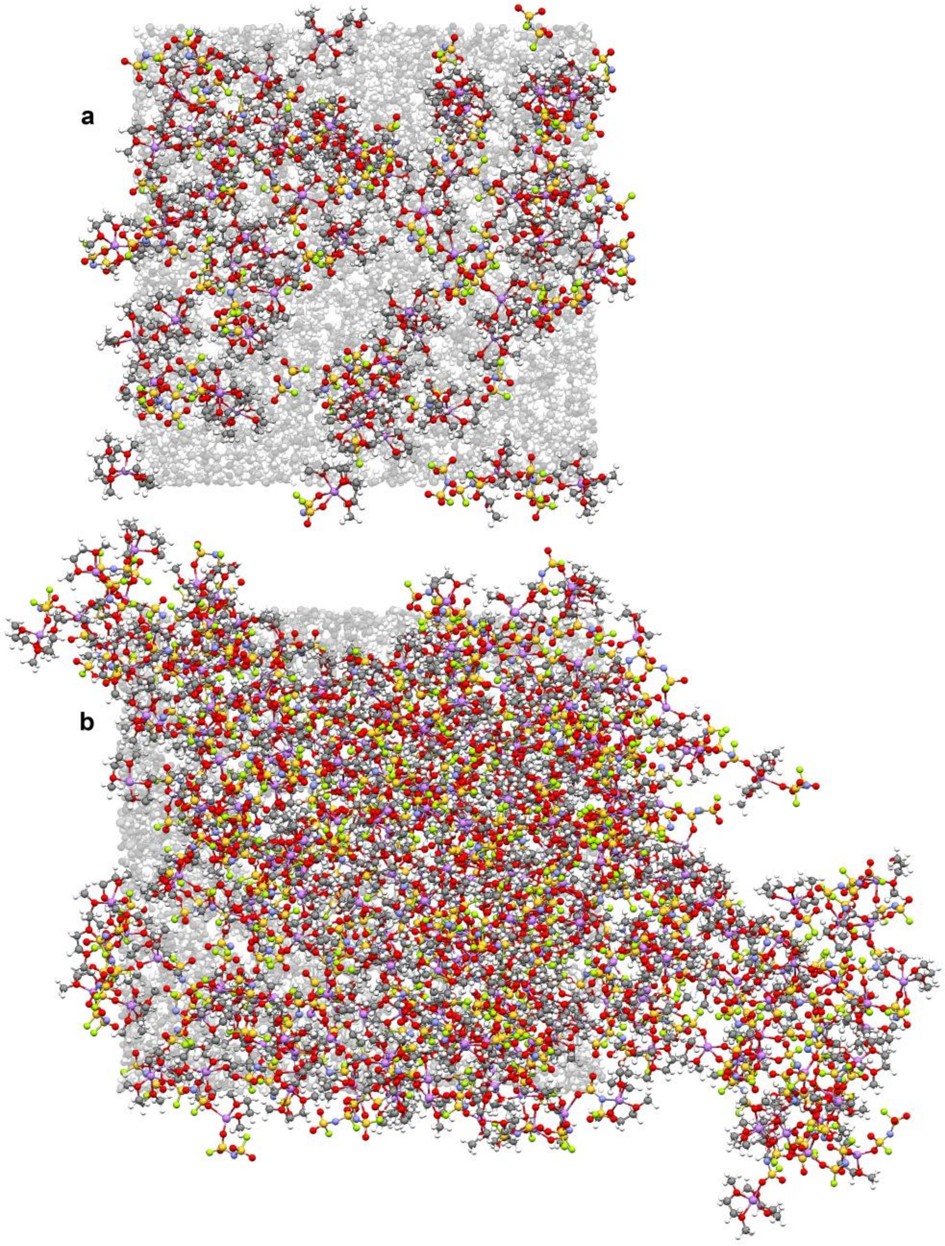

Supplementary Figure 10 | Two-dimensional snapshots of three-dimensional MD simulation boxes. (**a**) 1 M LiFSI-DME electrolyte. (**b**) 4 M LiFSI-DME electrolyte. Li-purple, O-red, N-blue, S-yellow, F-green, uncoordinated DME solvent and coordinated solvent/ions repositioned outside of the box with periodic boundaries-gray. The snapshots shown in this figure are analogous to a photograph of the simulation at a fixed point during the equilibrium runs when the solvent molecules and anions make and break bonds with the Li^+ cations thus resulting in a continuous evolution of the solvate species present (for the individual Li^+ cations)[1]. The gray shown in the figure is mostly due to coordinated solvent and ions extracted from the box and repositioned outside of the box so as not to cut the continuous solvates which extend across the periodic boundaries of the simulation box.

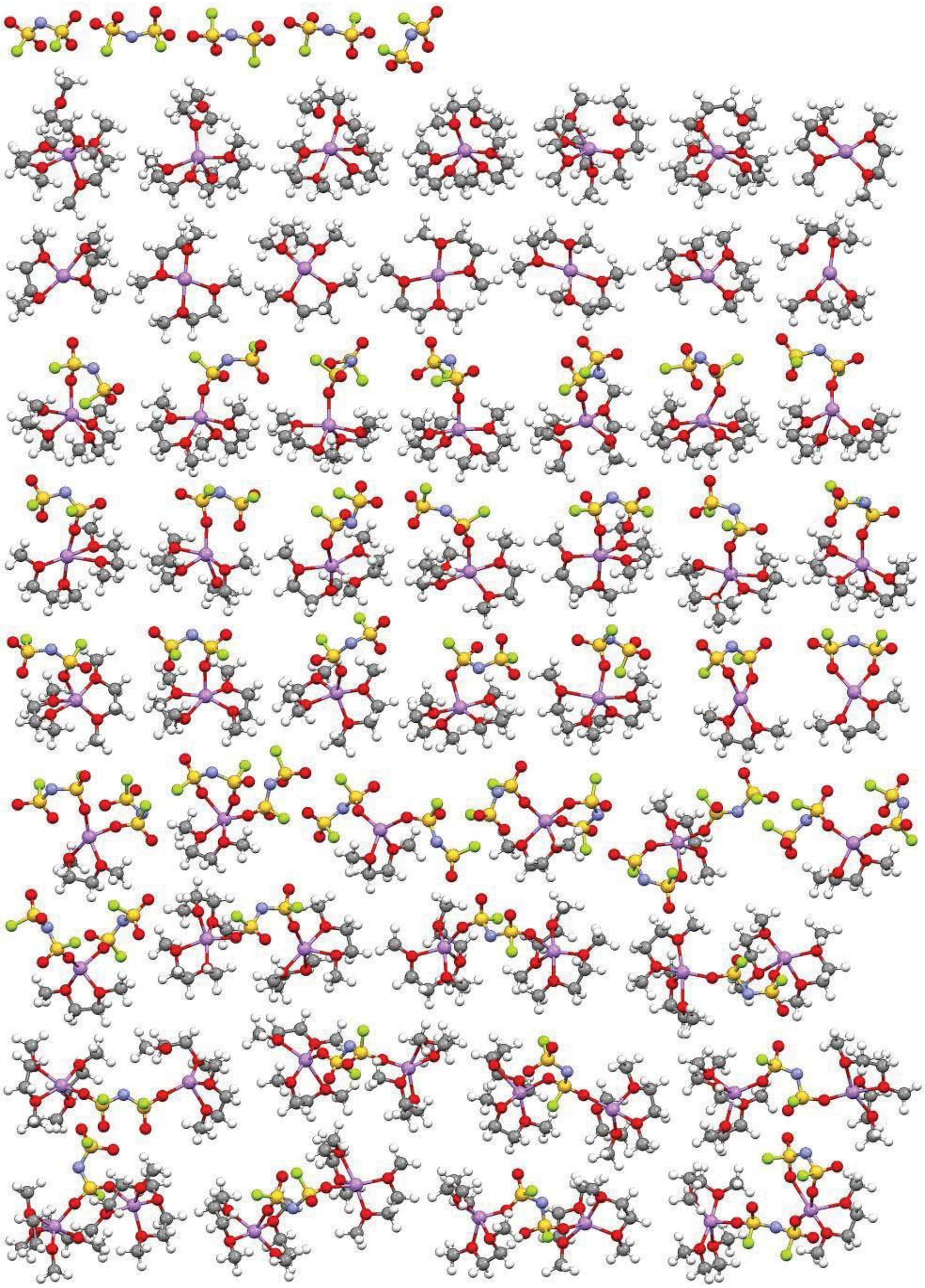

Supplementary Figure 11 | Solvates and uncoordinated anions extracted from the MD simulation snapshot of the 4 M LiFSI-DME electrolyte. Li-purple, O-red, N-blue, S-yellow, F-green.

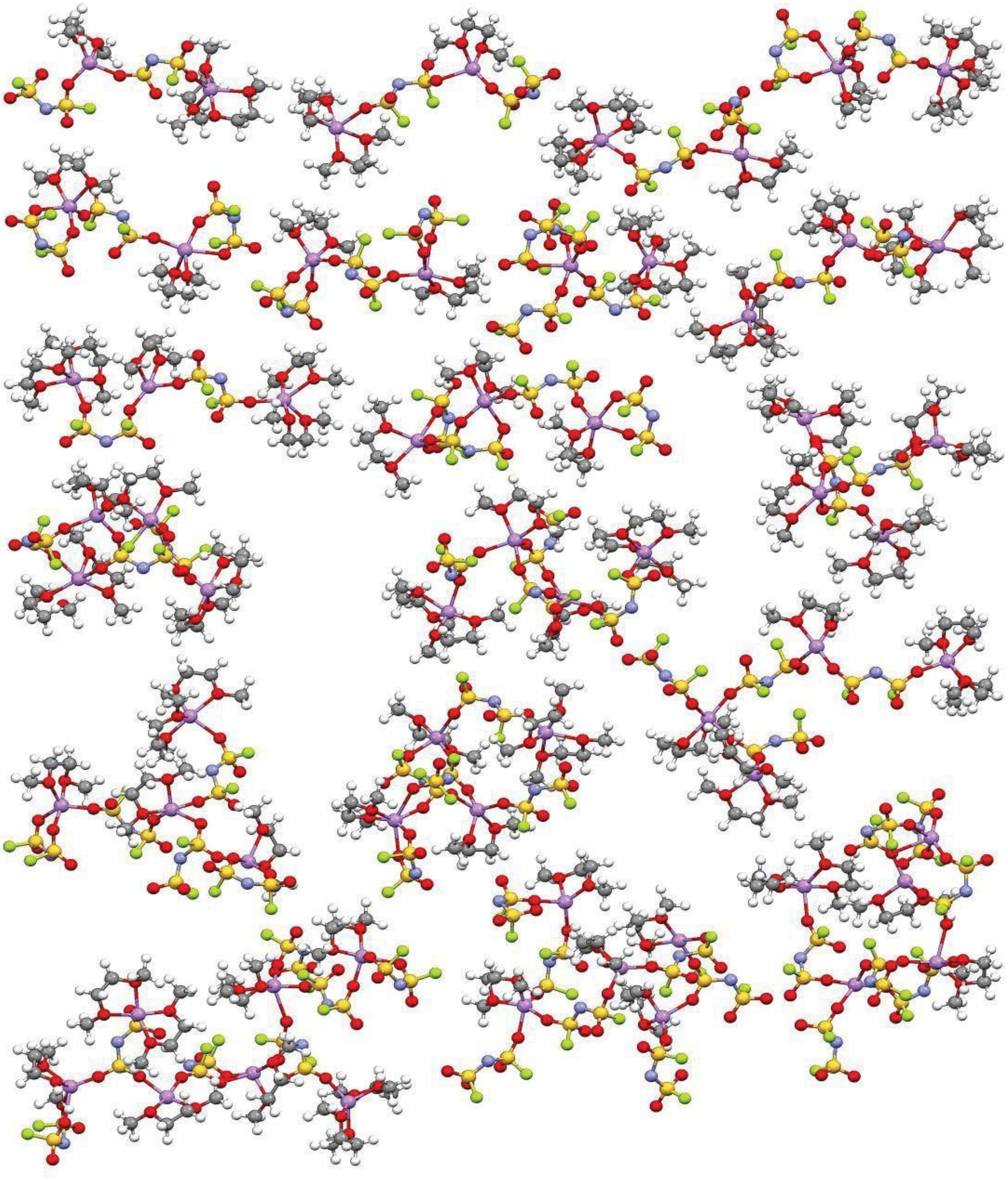

Supplementary Figure 11 (cont) | Solvates and uncoordinated anions extracted from the MD simulation snapshot of the 4 M LiFSI-DME electrolyte. Li-purple, O-red, N-blue, S-yellow, F-green.

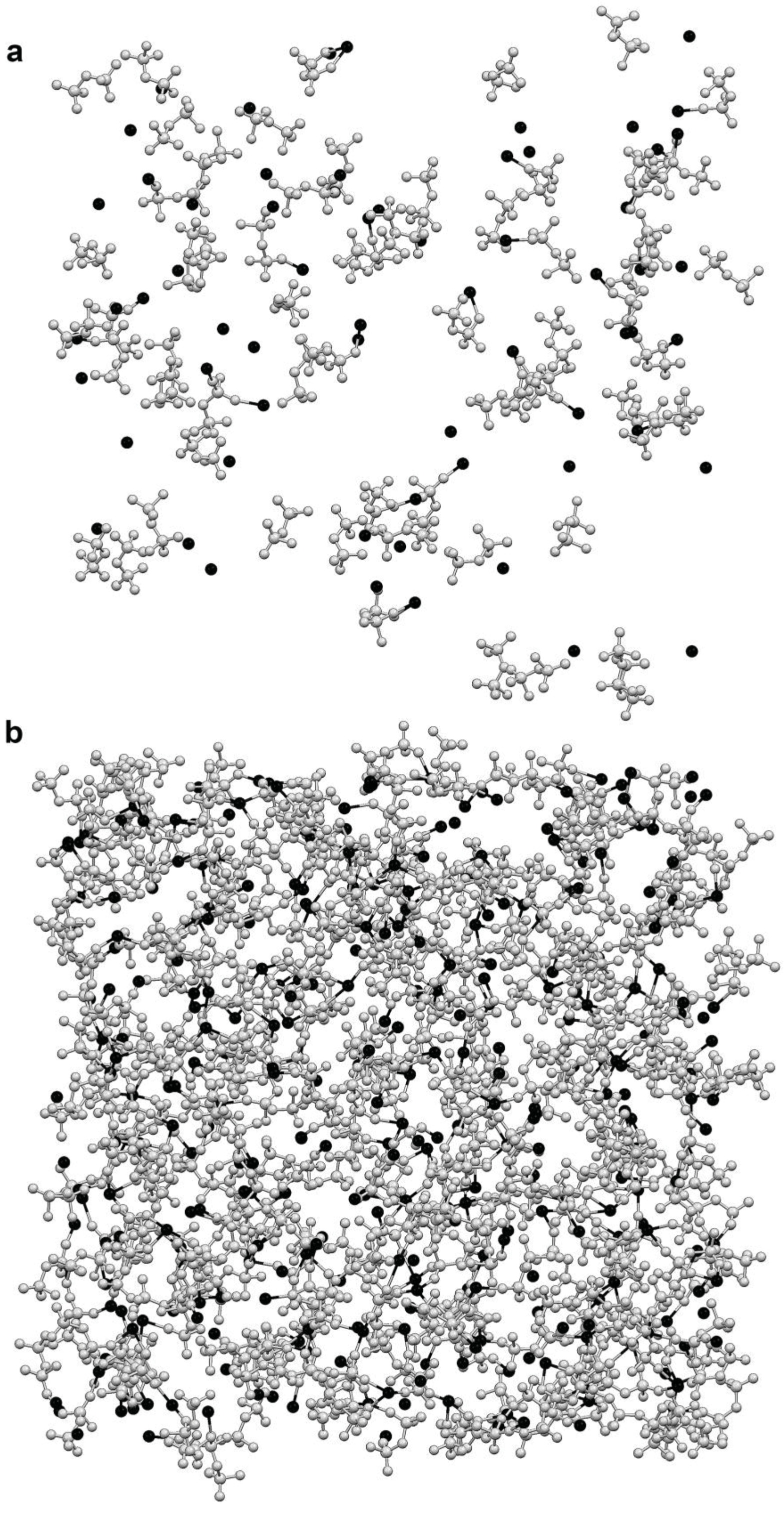

Supplementary Figure 12 | Ion distribution within the MD simulation box snapshots (solvent removed). (**a**) 1 M LiFSI-DME electrolyte. (**b**) 4 M LiFSI-DME electrolyte. Li-black, FSI^- anions-gray.

Supplementary Tables

Supplementary Table S1 | Concentration and conductivity data (at 25°C) for the LiFSI-DME electrolytes.

Concentration (mol L^{-1})	Mole Ratio (DME/LiFSI)	Conductivity (mS cm^{-1})
1 M	8.9/1	16.9
2 M	4/1	13.1
3 M	2.2/1	9.4
3.6 M	2/1	7.2
4 M	1.4/1	5.7
5 M	1/1	1.7

Supplementary Table 2 | XPS analysis (atomic %) for the Li surface plated on a Cu electrode using a 4 M LiFSI-DME electrolyte and a current density of 0.5 mA cm^{-2}.

Li deposited	O1s	S2p	F1s	C1s	N1s	L1s
Cycle 1	51.68	0.31	0.27	7.06	0.05	40.62
Cycle 10	36.20	7.22	6.14	14.86	3.64	31.94
Cycle 100	40.61	8.98	4.90	12.38	4.69	28.44

Supplementary Table 3 | MD simulation data for the LiFSI-DME at 60°C. The MD simulations of the 1 M and 4 M LiFSI-DME electrolytes at 60°C—rather the room temperature— are given to facilitate more rapid equilibration of the simulations. Note that the ionic association interactions of electrolyte tend to be strongly dependent on the salt concentration, but much less dependent upon the temperature[2,3] so [JQ1]the results below are believed to also be representative of the bulk electrolyte interactions at room temperature (the temperature used for the cell cycling). The simulation results indicate that over 60% of the Li^+ cations in the 1 M solution are fully solvated (on average) and thus uncoordinated to the anions. The majority of the anions in the 1 M solution are also uncoordinated as is also the case for much of the DME solvent present. In contrast, very little of the DME solvent is uncoordinated for the 4 M simulation. Nearly all of the ions in the 4 M electrolyte are coordinated—only about 3% of the anions are uncoordinated and 6% or so of the Li^+ cations are fully solvated producing a mixtures of both small and large solvated ion clusters.

Property	1 M	4 M
DME/LiFSI mole ratio	9.0/1	1.4/1
No. DME in MD box	576	448
No. LiFSI in MD box	64	320
Concentration (M) (from MD)	0.96	4.24
Simulation run length[a] (ns)	24.4 (6)	11.2 (8)
Simulation box length (Å)	48.02	50.05
Density (from MD) (g cm^{-3})	0.958	1.327
Conductivity (from MD) (mS cm^{-1})	23.2	4.2
Diff. Coeff. (from MD)-DME (kg m^{-3})	19.45	0.36
Diff. Coeff. (from MD)-FSI^- (kg m^{-3})	8.49	0.32
Diff. Coeff. (from MD)-Li^+ (kg m^{-3})	7.87	0.31
Fraction of free Li^+ ($r_{Li–N}$ > 4.75 Å)	0.62	0.06
Fraction of free FSI^- ($r_{Li–N}$ > 4.75 Å)	0.62	0.03
Fraction of free EOs ($r_{Li–O}$ > 2.40 Å)	0.75	0.08
Li^+ coordination numbers:		
# O (DME) (within 2.40 Å of Li^+)	4.42	2.60
# O (FSI^-) (within 2.40 Å of Li^+)	0.42	1.92

[a] NPT equilibration runs are shown in parentheses

"free Li^+" are fully solvated Li^+ cations (not coordinated to anions)
"free anions" are uncoordinated (naked) anions—anions are unsolvated in aprotic solvents such as DME
"free EOs" are uncoordinated ether oxygens from the solvent molecules (note that some of the solvent molecules have only one of the two ether oxygens coordinated—see figures below)

Supplementary References

1. Seo, D. M., Borodin, O., Balogh, D., O'Connell, M., Ly, Q., Han, S.-D., Passerini, S. & Henderson, W. A. Electrolyte solvation and ionic association III. Acetonitrile-lithium salt mixtures—transport properties. *J. Electrochem. Soc.* **160**, A1061–A1070 (2013).
2. Seo, D. M., Borodin, O., Han, S.-D., Ly, Q., Boyle, P. D. & Henderson, W. A. Electrolyte solvation and ionic association. I. Acetonitrile-lithium salt mixtures: Intermediate and highly associated salts. *J. Electrochem. Soc.* **159**, A553–A565 (2012).
3. Seo, D. M., Borodin, O., Han, S.-D., Ly, Q., Boyle, P. D. & Henderson, W. A. Electrolyte solvation and ionic association. II. Acetonitrile-lithium salt mixtures: Highly dissociated salts. *J. Electrochem. Soc.* **159**, A1489–A1500 (2012).
